# Harnessing Xylanase Potential in *Thermothelomyces fergusii*: Insights from Computational and Functional Analysis

**DOI:** 10.3390/jof11040250

**Published:** 2025-03-25

**Authors:** Abdul Waheed, Yi Chen, Ying Su, Yuxin Yan, Gang Liu

**Affiliations:** 1 Shenzhen Key Laboratory of Microbial Genetic Engineering, College of Life Sciences and Oceanography, Shenzhen University, Shenzhen 518060, China; waheed90539@gmail.com (A.W.);; 2 College of Physics and Optoelectronic Engineering, Shenzhen University, Shenzhen 518060, China

**Keywords:** ligand binding, dinitro salicylic acid, reducing sugar, hemicellulose

## Abstract

Xylanases are crucial for the breakdown of hemicellulose, enabling the conversion of lignocellulosic biomass into fermentable sugars for biofuels and other industrial applications. For the first time, we investigated the biochemical and genetic characteristics of 22 xylanase genes from *Thermothelomyces fergusii* within glycoside hydrolase (GH) families GH10, GH11, and GH43. Xylanase genes structural diversity clustered the phylogenetic tree into GH10, GH11, GH43-I, and GH43-II groups. Structural analysis revealed that all *TfGH10* and *TfGH11* genes contained conserved GH domains, with CBM1 present in *TfGH10-5* and *TfGH11-4*. Secondary domains, including CBM35, CBM42, and CBM91, were found in the GH43 gene family. The presence of key glutamic (Glu) and aspartic (Asp) residues in active sites is essential for substrate binding and catalysis. RT-qPCR analysis revealed substrate-dependent gene expression, with peak upregulation on day three in beechwood xylan (BWX) cultures and day two in corncob xylan (CCX) and rice straw (RS) cultures. Consistent with these findings, enzymatic assays demonstrated the highest xylanase activity in BWX-induced cultures, followed by RS and CCX, underscoring the differential regulation of these enzymes in response to distinct hemicellulosic substrates. These findings provide valuable insights into the structural, functional, and regulatory mechanisms of *T. fergusii* xylanases, facilitating their industrial application.

## 1. Introduction

Lignocellulosic biomass is the most abundant renewable carbon source on Earth, constituting approximately 40–50% cellulose, 25–35% hemicellulose, and 15–25% lignin, depending on the plant species and tissue type [1,2]. Hemicellulose, primarily composed of xylan, is a structural polysaccharide that strengthens plant cell walls by crosslinking cellulose and lignin [1]. The enzymatic deconstruction of lignocellulose into fermentable sugars is crucial for bioethanol production, animal feed improvement, and the paper and textile industries [3,4]. Among the various hydrolytic enzymes involved, xylanases are central to the breaking down of the β-1,4 bond in xylan, converting into xylo-oligosaccharides and xylose [5]. Xylanases are categorized into glycoside hydrolase (GH) families, with GH10, GH11, and GH43 being the most extensively studied due to their industrial relevance and distinct catalytic properties [6]. GH10 enzymes degrade complex xylan [7], GH11 targets linear xylans in acidic conditions [8], and GH43 includes β-xylosidases and α-arabinofuranosidases, which act synergistically with GH10 and GH11 to enhance hydrolysis [9,10]. Carbohydrate-binding modules (CBMs) facilitate substrate binding and enzymatic efficiency. Usually, CBM1, found in many GH10 and GH11 xylanases, targets insoluble lignocellulose [8,11]. Meanwhile, the GH43 family contains diverse CBMs, such as CBM35, CBM42, and CBM91, which help enzymes recognize and bind to substrates, improving their efficiency in breaking down arabinoxylan [10].

The genomic and transcriptomic characterization of xylanase genes has expanded our understanding of lignocellulose degradation. Transcriptomic profiling of the *Fusarium commune* revealed extensive cellulolytic and hemicellulolytic enzyme production, including GH10 and GH11 xylanases, which were differentially regulated depending on substrate availability [12]. Functional genomic studies of *Neurospora crassa* demonstrated that the GH10, GH11, and GH43 enzymes act synergistically to degrade xylan into fermentable sugars [13]. Additionally, the xylanase repertoire of *Aspergillus fumigatus*, analyzed through sequence-based thermostability prediction, revealed several enzymes with potential applications [14]. Similarly, a GH10 xylanase from *Sporisorium reilianum* exhibited unique structural adaptations, highlighting evolutionary divergence in xylanase families [15].

In the world of xylanases, Asp and Glu are key players in the breakdown of glycosidic bonds, where they act as proton donors and nucleophiles in the GH10, GH11, and GH43 families [13,14,16]. In addition to in silico and in vitro characterizations, these enzymes are crucial for industrial applications, especially when expressed in suitable microbial hosts like *Aspergillus niger*, *Pichia pastoris*, *Saccharomyces cerevisiae*, and *Escherichia coli*, chosen for their ease of genetic manipulation and scalability in bioengineering [5,13,17,18]. A GH11 xylanase from a saline–alkali soil bacterium, expressed in *P. pastoris*, demonstrated remarkable stability under extreme pH and salinity, making it a robust candidate for industrial applications [19]. Similarly, a few *GH10* and *GH11* genes of *N. crassa* were successfully expressed in *E. coli* and functionally characterized, required for the glycosylation of xylo-oligosaccharides [13]. Recent advancements, such as codon optimization, have enhanced xylanase efficiency and catalytic activity [20]. Structural studies, including X-ray crystallography and quantum mechanics/molecular mechanics (QM/MM) simulations, have revealed diverse mechanisms in GH43 xylanolytic enzymes, improving substrate specificity [16]. Additionally, an extensive number of potentially active xylanase genes were identified within the transcriptome and genome analysis of filamentous fungi, including *Myceliophthora thermophila*, *Malbranchea cinnamomea*, *N. crassa*, *A. niger*, and *Trichoderma reesei* [13,21,22,23].

Integrating genomics, proteomics, and structural biology will continue refining our understanding of xylanase function and optimization. Bioengineering efforts, including directed evolution and site-directed mutagenesis, are expected to enhance the thermal and alkaline stability of GH10, GH11, and GH43 xylanases, broadening their industrial applications [24]. Applying multi-enzyme cocktails incorporating CBM-containing xylanases will further improve the degradation efficiency of lignocellulosic biomass [25]. As global demand for sustainable bioproducts increases, GH10, GH11, and GH43 xylanases will play a critical role in the biofuel production, food processing, and biorefinery industries. Future research should focus on optimizing enzyme formulations and reaction conditions to enhance the economic feasibility of industrial xylanase applications.

Despite the industrial significance of xylanases, the genomic and functional characteristics of these enzymes in *Thermothelomyces fergusii* (CBS 405.69) remain underexplored. *T. fergusii*, previously classified as *Corynascus thermophilus*, is a thermophilic member of the Chaetomiaceae family, closely related to *Myceliophthora fergusii*. The fungus thrives optimally at 45 °C, with a broader growth range between 22 and 55 °C [26]. The species was collected from mushroom compost made up of equal proportions of hay, manure, corncobs, and wheat straw [27]. *T. fergusii* is a promising fungal species known for its extensive repertoire of lignocellulose-degrading genes. The genome of this fungus abundantly encodes 49 genes from the auxiliary activity (AA) family, 170 genes from the glycoside hydrolase (GH) family, and 70 genes from the glycosyl transferase (GT) family, enabling the organism to efficiently degrade complex polysaccharides, such as cellulose and hemicellulose. Identifying and characterizing these lignocellulolytic genes in *T. fergusii* have significant implications for industrial applications, particularly in biofuel production and biomass valorization.

To address this knowledge gap, we conducted a detailed analysis of the *GH10*, *GH11*, and *GH43* gene families in the *T. fergusii* genome to elucidate their structural features and functional relevance, with special emphasis on industrial applications. In this study, we employed comprehensive bioinformatic approaches to investigate the *GH10*, *GH11*, and *GH43* genes from *T. fergusii*, aiming to interpret their roles in hemicellulose degradation. Concurrently, biochemical analyses, including assessments of biomass production, protein secretion, and enzymatic activity, were performed on fungal secretomes exposed to hemicellulosic substrates: corncob xylan (CCX), beechwood xylan (BWX), rice straw (RS), and xylose (XLS). Additionally, we examined the expression profiles of these gene families under hemicellulose-rich conditions to provide deeper insights into their functional roles in polysaccharide hydrolysis.

## 2. Materials and Methods

### 2.1. Screening of GH10, GH11, and GH43 Genes

Genome sequences of *Myceliophthora thermophila* (*Sporotrichum thermophile*) *v2.0* and *Malbranchea cinnamomea* CBS 34,355 were downloaded from the MycoCosm JGI database, while *N. crassa* OR74A genome (assembly NC12) was downloaded from the NCBI database. The *T. fergusii* whole-genome sequence was obtained from the JGI MycoCosm database for the genomic identification of the *GH10*, *GH11*, and *GH43* gene families [26]. The protein sequences of GH10, GH11, and GH43 of these strains are provided in Supplementary Table S1. For the screening of GH10, GH11, and GH43 homologous proteins in all four fungi, a reference Pfam—PF00331 for GH10, pfam00457 for GH11, and PF04616 for GH43—was found in the InterPro database, and their homologous Pfams were computed in the Pfam web server (http://pfam.xfam.org/, accessed on 30 October 2024). We gave the names of all proteins according to their genomic and protein sequence number. We added the genus and species name of each fungus before the name of their gene family: for example, for the *M. thermophila* gene families, these were MtGH10, MtGH11, and MtGH43, and, similarly, for the *M. cinnamomea* gene families, the names were McGH10, Mc GH11, and McGH43. Moreover, the *N. crassa* gene families were renamed NcGH10, NcGH11, and NcGH43, while the *T. fergusii* gene families were renamed TfGH10, TfGH11, and TfGH43. To identify the TfGH10, TfGH11, and TfGH43 homologous protein sequences, TB tools-II (Toolbox for Biologist) v2.152 [28] was used to achieve the BLASTp (Basic Local Alignment Search Tool for Protein) in default mode, using PF00331 for GH10, PF00457 for GH11, and PF04616 for GH43, their homologous generated file, and the whole-genome protein.

### 2.2. Construction of Phylogenetic Tree of TfGH10, TfGH11, and TfGH43 Gene Families

Phylogenetic analyses were conducted using the GH10, GH11, and GH43 proteins from *M. thermophila*, *N. crassa*, *M. cinnamomea*, and *T. fergusii*. The dataset included five TfGH10, five TfGH11, twelve TfGH43 proteins, and forty-one well-characterized proteins from the other three fungal species. Multiple-sequence alignment (MSA) was performed in the Molecular Evolutionary Genetics Analysis (MEGA-11) software using the MUSCLE algorithm with parameters set to gap open = −2.90 and gap extend = 0.00. A neighbor-joining (NJ) phylogenetic tree was constructed in MEGA-11, with 1000 bootstrap replicates [29]. The Nevik data file was subsequently generated and submitted to the iTOL v5 free online tool for final validation of the phylogenetic tree [30]. Additionally, the amino acid (AA) sequence length, CDS length, total exon-to-intron ratio counts, and scaffold/chromosome numbers were determined using TBtools-II v2.152 [28].

### 2.3. Comprehensive Gene Structure Analysis of TfGH10, TfGH11, and TfGH43 Genes

The amino acid sequences of all twenty *TfGH10*, *TfGH11*, and *TfGH43* genes were analyzed using MEME Suite v5.5.4 to identify conserved motifs [31]. The genome annotation data from the original GFF file were processed to determine the gene structure of *TfGH10*, *TfGH11*, and *TfGH43.* Conserved-domain analysis was conducted by searching against the Pfam database in NCBI-CDD (Conserved Domain Database), generating a corresponding hit-data file [32]. Full-length protein sequence alignment was performed using the MUSCLE algorithm in MEGA-11, and the Nevik file was computed using the methods designated above. The entire analysis was conducted using TBtools v2.152 software.

### 2.4. Building of Protein 3D Structure and Protein–Ligand Interaction

Three-dimensional (3D) models for all 22 xylanase proteins were constructed utilizing the free repository AFPD (Alph-Fold Protein Database) [33]. Sequence identity (SI) and sequence similarity (SS) were determined based on the pairwise analysis of *T. fergusii* proteins and AlphaFold-predicted protein models using the online EMBOSS Stretcher tool (https://www.ebi.ac.uk/jdispatcher/psa/emboss_stretcher, accessed on 26 December 2024). Bio-edit-7.2 software was used to construct the protein homology and conserved residues analysis of TfGH10, TfGH11, TfGH43, and other fungi xylanase proteins. The MIB2 database (http://combio.life.nctu.edu.tw/MIB2/, accessed on 30 December 2024) was run to build the protein structure models, including 3u7bA, 5juhA1, and 4peuA0 for TfGH10-4, TfGH11-1, and TfGH43-6, respectively. First, we used the Discovery Studio Software (DSS-2021) to clean the ligand and water molecules and then saved the clean PDB structure [34]. We downloaded the PDB structure of (1,4)-beta-xylotetraose (ID:5289594) and (1,4)-beta xylohexaose (ID:74539951) from the PubChem database [35]. We used the PyRX-1.1 software to interact ligands with a protein structure and then saved the best model based on the binding affinity for further analysis [36]. Moreover, we used DSS to visualize the interaction sites, catalytic amino acids, protein 2D, and 3D structure. Distances between catalytic residues were measured using PyMOL software-4.6.0 [37].

### 2.5. Analysis of Gene Location, Duplication, Circos, and Gene Mutation

To identify the genomic positions and protein sequences of the *TfGH10*, *TfGH11*, and *TfGH43* genes, the distribution of these xylanase genes across scaffolds/chromosomes was examined using TBtools v2.152. The visualization of *TfGH10*, *TfGH11*, and *TfGH43* gene locations on the scaffolds of *T. fergusii* was conducted with the online tool MapGene2Chromosome v2 [38]. Gene duplication events and the Ka (nonsynonymous)/Ks (synonymous) ratios were analyzed using advanced circose analysis and computed in TBtools v2.152. Specifically, scaffold ID with their length, genome feature file, and linked gene information files were uploaded and optimized the circos. These analyses were further used to identify segmental and tandem duplication of the *TfGH10*, *TfGH11*, and *TfGH43* genes [39].

### 2.6. Analysis of Gene Enrichment and Hypothetical Model of Xylanase Enzyme Function

Gene ontology (GO) enrichment analyses were conducted by obtaining the GO-basic ontology data from the online repository (http://purl.obolibrary.org/obo/go/go-basic.obo, accessed on 28 November 2024), while the gene Query2 Go data were retrieved from the MycoCosm (https://mycocosm.jgi.doe.gov/mycocosm/home, accessed on 28 November 2024). Moreover, enzyme commission (EC) numbers were screened from KEGG files to predict the functional properties of xylanase enzymes. The molecular structures of xylohexaose (X6), xylotriose (X3), xylobiose (X2), and xylose were obtained from the PubChem database [35]. Based on these data, a proposed enzymatic model was developed, indicating that the *TfGH10* and *TfGH11* genes (EC: 3.2.1.8, endo-1,4-β-xylanase) preferentially cleave internal β-1,4-glycosidic linkages in xylan, whereas the *TfGH43* genes (EC: 3.2.1.37, xylan 1,4-β-xylosidase) hydrolyze the non-reducing ends of xylo-oligosaccharides, side arabinose chain, or xylobiose units. GO enrichment analyses file and structural diagrams for all xylanase genes were performed in TBtools v2.152.

### 2.7. Fungal Strain Culturing Condition

The thermophilic fungus *T. fergusii* CBS 405.69 was obtained from the Westerdijk Fungal Biodiversity Institute, Netherlands, and utilized for extensive genomic, biochemical, and gene expression analyses. Corncob xylan (CCX), beechwood xylan (BWX), and xylose (XLS) were procured from Sigma-Aldrich (Darmstadt, Germany), while rice straw (RS) was purchased through an online platform and used as a carbon source. RS was pretreated by soaking in 1.5% NaOH to remove lignin, followed by sterilization in an autoclave at 121 °C for 20 min. Afterward, it was washed with distilled water to adjust the pH to approximately 7, then dried in an oven, and ground into a powdered form [40]. *T. fergusii* was first cultured on PDA (potato dextrose agar) at 45 °C for two to three days. The spores were subsequently sieved through sterilized filter paper, and the spore density was calibrated to 7 × 10⁷ spores mL^−1^, as stated in a published article [40]. The spores were cultured in a liquid seed medium at 45 °C for one day. The seed medium (total volume: 1 L) contained 100 mL Mendel nutrient salts [(NH_4_)_2_SO_4_, urea, KH_2_PO_4_, MgSO_4_·7H_2_O], 1 mL trace elements (FeSO_4_·7H_2_O, ZnSO_4_·7H_2_O, MnSO_4_·H_2_O/MnCl_2_), citrate buffer 50 mL (1M, pH 4.5), tryptone (1 g), Tween-80 (1.5 mL), CaCl_2_·2H_2_O (0.4 g), and glucose (20 g). All reagents, excluding CaCl_2_·2H_2_O and glucose, were dissolved in 800 mL of distilled water with continuous shaking. The remaining 200 mL contained CaCl_2_·2H_2_O, and glucose was added after autoclaving at 121 °C for 20 min. The seed culture medium was inoculated with around 500 μL of spore suspension and cultivated at 45 °C in a shaker at 150 rpm for 24 h.

Liquid fermentation broth was prepared by adding Mendel nutrient salts (100 mL), citrate buffer (50 mL), tryptone (20 g), yeast extract (5 g), Tween-80 (0.5 mL), and CaCl_2_·2H_2_O (0.4 g). After 24 h of seed culture growth, 5 mL of mycelial suspension was transferred to 50 mL of fermentation medium (excluding glucose) supplemented with either 2% CCX, BWX, XLS, or 1 g of RS as a carbon source. Culture samples were kept at 45 °C in a shaker at 150 rpm for five days, with daily sample collection for biomass quantification, protein estimation, and enzymatic activity measurements. The culture broth and fungal biomass were isolated by centrifugation at 6000 rpm for 10 min at 4 °C, and the supernatants were preserved at −80 °C for further analysis. RNA extraction was performed under the same cultivation conditions and carbon source concentrations as those used for supernatant collection. Mycelia were harvested by filtering the liquid culture through 0.6 μm filter paper. RNA was extracted from mycelial samples collected on days 2–5. After measuring RNA concentrations, optimal RNA concentrations were observed on days 2 and 3. Consequently, samples from these two time points were selected for further gene expression analysis.

### 2.8. Biochemical Analysis

Fungal biomass was quantified by measuring the dry mycelial weight. Mycelial pellets were obtained through centrifugation, as described above, and were dried in an oven at 60 °C until a constant weight was attained. Protein concentrations in the filtered secretome were quantified using the Bradford assay, following the previously published protocol [41]. Briefly, 0.02 mL of protein solution was mixed with 0.2 mL of Bradford reagent and incubated at room temperature. A standard curve was generated using BSA (bovine serum albumin) at different concentrations, namely 0, 0.025, 0.050, 0.075, 0.1, and 0.125 mg/mL. Absorbance was measured at 595 nm, and the protein content was calculated by interpolating the values from the standard calibration curve.

Xylanase activity and reducing sugar concentrations were quantified using the dinitro salicylic acid (DNS) method. The xylan substrate solution was prepared by dissolving 1% birchwood xylan (Sigma-Aldrich) in 0.05 M citrate buffer (pH 5.3). Enzymatic reactions were conducted at 50 °C for 20 min by mixing 0.15 mL of 10-fold-diluted xylanase enzyme from the culture supernatant with 0.5 mL of xylan solution. After incubation, 0.9 mL of DNS solution was added to the sample, and the resultant mixture was heated for 5 min. The reaction solution was subsequently cooled on ice for a few minutes, and the optical density (OD) was recorded at 540 nm using an ultraviolet spectrophotometer (Puyuan Instruments, Ltd., Shanghai, China). A calibration curve was constructed using 0, 0.25, 0.5, 0.75, 1, and 1.5 mg/mL xylose concentrations. Data analysis was performed according to the DNS assay protocol previously reported [42].

### 2.9. RNA Extraction and Expression Analysis

Total RNA was extracted from the collected mycelia using the Trizol method [43]. cDNA synthesis was carried out using the HiScript III SuperMix cDNA Synthesis Kit (Nanjing Vazyme Biotech Co., Ltd., Nanjing, China) according to the manufacturer’s protocol. The gene-specific primers used for target gene amplification are listed in the Supplementary File (Supplementary Table S2). Quantitative real-time PCR (qRT-PCR) was performed in 96-well plates with a total reaction volume of 20 μL per gene. Each reaction mixture contained 10.5 μL of Hieff UNICON^®^ Universal Blue qPCR SYBR Green Master Mix (Yeasen Biotechnology, Shanghai, China), 0.4 μL of each forward and reverse primer (final concentration: 2.5 μM), 1.2 μL of diluted cDNA (10 ng), and 7.5 μL of nuclease-free water. qRT-PCR was performed on a qTOWER^3^ G system (Jena, Germany). The thermal cycling conditions included an initial denaturation at 95 °C for 2 min, followed by 40 cycles of denaturation at 95 °C for 10 s, annealing at 60 °C for 30 s, and extension at 60 °C for 1 min. Each reaction included at least three biological replicates to ensure data reliability. The *GAPDH* (glyceraldehyde 3-phosphate dehydrogenase) internal reference gene was used for the normalization analysis. One-way ANOVA was performed in GraphPad Prism-9 software to assess the significant differences between the control and treated samples.

## 3. Results

### 3.1. General Features of GH10, GH11, and GH43 Genes

Five *GH10*, five *GH11*, and twelve *GH43* genes were identified from the *T. fergusii* genome. The *GH10* and *GH11* genes were nominated with IDs based on the initial letters of the genus (*T*), species (*f*), and their chromosomal location, whereas the *GH43* gene’s ID was assigned according to the sequence order of its proteins (Table 1). The genome of *T. fergusii* comprises 247 scaffolds (Sc) with 22 xylanase genes distributed across 14 scaffolds (Table 1). Among these, the shortest and longest genomic DNA (gDNA) sequences were identified as *TfGH11-2* and *TfGH43-10*, with lengths of 785 bp and 1785 bp, respectively. The coding sequence (CDS) lengths of xylanase genes ranged from 510 bp (*TfGH10-2*) to 1785 bp (*TfGH43-10*), translating to protein sizes varying between 170 and 595 amino acids. Signal peptides (SPs) were detected in all five *TfGH10* and *TfGH11* genes but were lacking in four *TfGH43* genes. The lengths of the signal peptides for all genes are detailed in Table 1. A secondary catalytic-binding module (CBM1) was identified in *TfGH10-5* and *TfGH11-4*, whereas CBM91 was observed in *TfGH43-2*, *TfGH43-4*, *TfGH43-6*, and *TfGH43-10*. Additionally, CBM35 was found in TfGH43-3 and TfGH43-5, while CBM42 was uniquely detected in TfGH43-11 (Table 1). Eight of the discovered genes were missing introns, and the exon–intron (E:I) ratios for all 22 genes are presented in Table 1.

### 3.2. Phylogenetic Association of TfGH10, TfGH11, and TfGH43 Family Members

**Figure 1:**
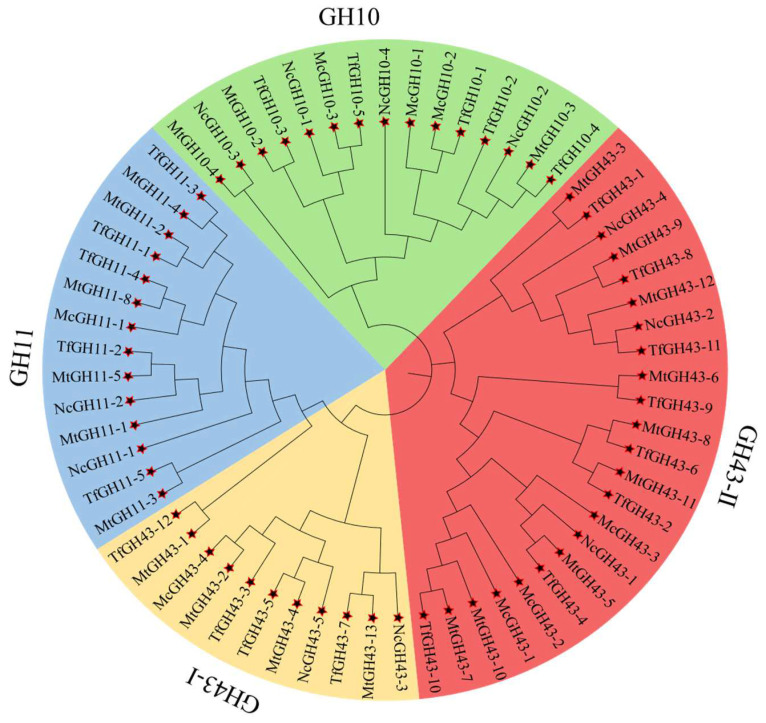
Phylogenetic tree of GH10, GH11, GH43 xylanase families from *M. thermophia* (Mt), *N. crassa* (Nc), *M. cinnamomea* (Mc), and *T. fergusii* (Tf), *representing* four fungal species. GH10 and GH11 proteins are represented in light green and light blue colors, respectively. TheGH43 family is further divided into two subclades, GH43-I (light yellow) and GH43II (light red), based on the protein variations linked to a secondary non-catalytic domain.

To investigate the relationship between the *GH10*, *GH11*, and *GH43* genes from *T. fergusii* and those of three other fungal species, a neighbor-joining (NJ) phylogenetic tree was constructed. The analysis was performed based on multiple-sequence alignments of xylanase protein sequences, including five TfGH10, five TfGH11, and twelve TfGH43 proteins from *T. fergusii*, together with forty-one known xylanase proteins from *M. thermophila*, *N. crassa*, and *M. cinnamomea*. These included three MtGH10, six MtGH11, and thirteen MtGH43 from *M. thermophila*; four NcGH10, two NcGH11, and five NcGH43 from *N. crassa*; and three McGH10, one McGH11, and four McGH43 from *M. cinnamomea*. The phylogenetic analysis grouped the xylanase genes into four distinct clades: GH10 (light green), GH11 (light blue), GH43-I (yellowish brown), and GH43-II (red). Due to the presence of short domains, McGH43-5, MtGH10-1, MtGH43-6, and MtGH43-7 were excluded from the analysis (Supplementary Table S1). The clustering pattern revealed that the GH43 proteins were positioned between the GH10 and GH11 groups, highlighting their evolutionary relationship. Additionally, proteins containing secondary domains, such as CBM35 and CBM91, were grouped in adjacent branches, supporting their functional and evolutionary association (Figure 1).

### 3.3. Gene Structure, Conserved Domain, and Motif Analysis of TfGH10, TfGH11, and TfGH43

**Figure 2:**
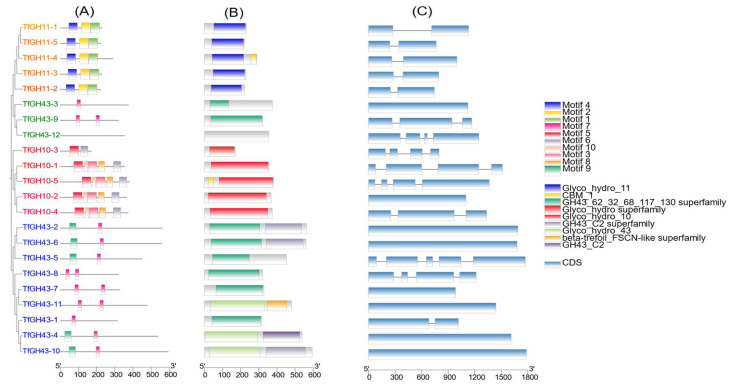
Full gene structure analysis of the TfGH10, TfGH11, and TfGH43 proteins in *Thermothelomyces fergusii*: (**A**) box-like, ten different colors represent the ten conserved motifs; (**B**) the conserved domain red color is Glyco-hydro-10 (GH10), the blue color is Glyco-hydro-11 (GH11), and the yellow color is representing the CBM1 domain. GH43 with superfamily 62, 32, 68, 117, and 130 are dark green, while light green represents the Glyco-hydro-43 (GH43) family. Moreover, the TfGH43-2 and TfGH43-6 secondary domain (GH43_C2 superfamily or CBM91) are shown in a gray color, while the TfGH43-4 and TfGH43-10 secondary domain (GH43_C2 or CBM91) is represented in light purple color. Additionally, the orange color represents beta-trefoil (CBM42). (**C**) Exon or CDS (light blue box) and intron (black lines).

Homology modeling was conducted using sequence alignment and gene structure analysis to investigate the activity and potential functions of the *TfGH10*, *TfGH11*, and *TfGH43* genes. The MEME program identified 10 conserved motifs (motifs 1–10) across the 22 xylanase genes. Specifically, motifs 1, 2, and 4 were the most prevalent among the TfGH11 proteins, whereas motifs 3, 5, 6, 8, and 10 were common among the TfGH10 proteins. Interestingly, although the TfGH43 protein sequences were the longest, only motifs 7 and 9 were exclusive to this group (Figure 2A). Motif LOGOs were constructed using the MEME online tool (Supplementary Figure S1). Motifs 1, 2, 3, and 5 exhibited higher consensus sequence lengths (50 amino acids), while motifs 7 and 10 showed shorter sequences (20 amino acids) (Supplementary Figure S1). Conserved-domain analysis revealed that the GH10 domain was present in all five *TfGH10* genes, while CBM_1 was specific to *TfGH10-5* (Figure 2B). Similarly, the GH11 domain was conserved across all five *TfGH11* genes, with CBM_1 detected exclusively in TfGH11-4 (Figure 2B). The conserved domains within the TfGH43 family displayed significant diversity. Among these, eight TfGH43 proteins contained GH43 domains linked to superfamilies 62, 32, 68, 117, and 130. The Glyco-hydro 43 domain was observed in three TfGH43 proteins, whereas TfGH43-12 lacked any conserved domain (but the JGI database revealed that TfGH43-12 belongs to GH43, the glycoside hydrolase family 43). The secondary-domain analysis based on the NCBI Conserved Domain Database (CDD) indicated the presence of the GH43_C2 superfamily in TfGH43-2 and TfGH43-6, while TfGH43-4 and TfGH43-10 belonged to GH43_C2. The JGI database identified these secondary domains as CBM91 for these four genes (Table 1). Additionally, TfGH43-11 contained a beta-trefoil-FSCN-like superfamily domain in NCBI CDD search or CBM42 in the JGI database (Figure 2B and Table 1). The structural analysis of the genomic DNA (gDNA) revealed an exon–intron (E:I) relation in the 22 xylanase genes. Mainly, eight genes lacked introns, while TfGH43-5 had the highest complexity, with five exons and four introns (Figure 2C).

### 3.4. Prediction of Protein 3D Model and Catalytic Active Sites

**Figure 3:**
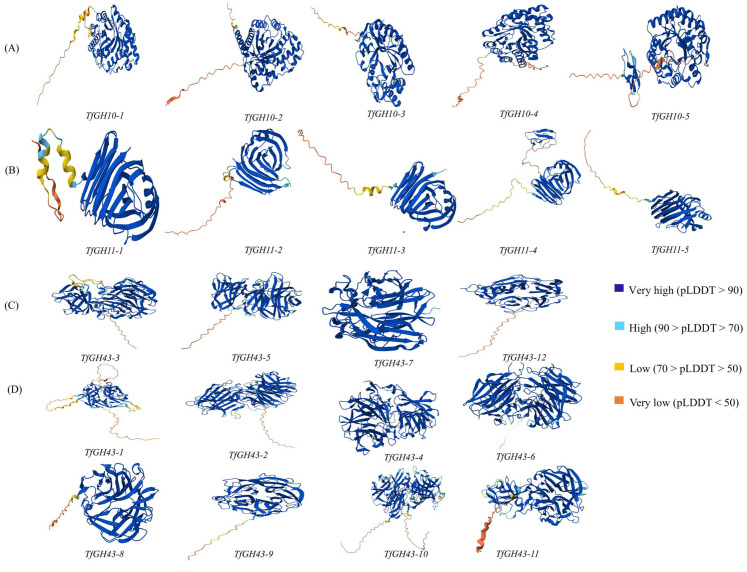
AlphFold database predicted the 3D models of proteins based on the high similarity and positivity of residues: (**A**) GH10; (**B**) GH11; (**C**) GH43-I; and (**D**) GH43II. pLDDT is the per residue model confidence score (pLDDT), from 0 to 100.

The AlphaFold Protein Database (AFDB) was employed to predict the three-dimensional structures of proteins, with high-identity templates used for modeling the TfGH10, TfGH11, and TfGH43 proteins (Figure 3 and Supplementary Table S3). Based on phylogenetic tree analysis, we divided the protein 3D structures into four distinct groups. For TfGH10, templates A0A175VX36, V5JB86, G2QG07, G2QGN6, and G3FAQ8 were used to model TfGH10-1, TfGH10-2, TfGH10-3, TfGH10-4, and TfGH10-5, respectively (Figure 3A and Supplementary Table S3). Similarly, the three-dimensional structures of TfGH11-1, TfGH11-2, TfGH11-3, TfGH11-4, and TfGH11-5 were constructed using templates G2QDB9, Q2HI25, G2Q4S6, A0A0B5JC15, and G2QIK8, respectively (Figure 3B and Supplementary Table S3). Within the TfGH43 group, four proteins (TfGH43-3, TfGH43-5, TfGH43-7, and TfGH43-12) were categorized into the TfGH43-I sub-group, with their corresponding 3D models based on templates G2Q562, G2QDD9, G2R299, and G2QHQ9 (Figure 3C and Supplementary Table S3). Additionally, templates G2Q7W6, G0RY67, G2QDZ0, G2QCC8, G2QFK1, G2QFK0, G2QAJ6, and Q2H847 were used to predict the 3D structures of TfGH43-1, TfGH43-2, TfGH43-4, TfGH43-6, TfGH43-8, TfGH43-9, TfGH43-10, and TfGH43-11, respectively (Figure 3D and Supplementary Table S3). The predicted protein SI for TfGH10 ranged from 44.6% to 87.2%, with the lowest value attributed to the shorter sequence of TfGH10-3 and the highest to TfGH10-4. The SS range for TfGH10 was between 47.7% and 93.6%. Similarly, the predicted SI and SS ranges for TfGH11 were 73–94.8% and 80.2–96.9%, respectively (Table 1). For TfGH43, the protein SI ranged from 55.2% to 97%, while the SS range was 60 to 99.1% (Table 1). These findings suggest that the xylanase genes across different genomes may share a common ancestral origin. However, structural and functional variations may have emerged over time, potentially contributing to their divergence.

**Figure 4:**
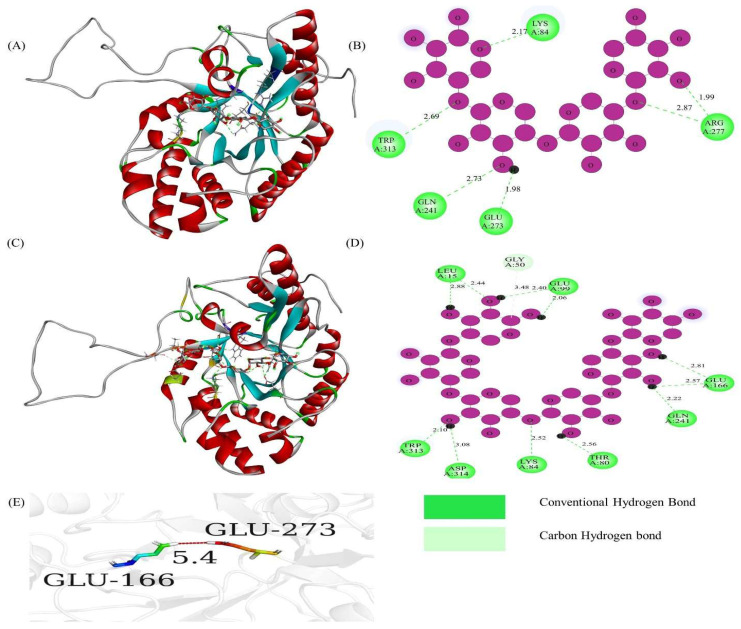
Three-dimensional and two-dimensional structural analysis of TfGH10-4 and its ligand interactions: (**A**) three-dimensional structure interaction with xylotetraose; (**B**) two-dimensional structure showing the interaction of active site residues and Glu-273, a catalytic residue, with xylotetraose; (**C**) three-dimensional structure interaction with xylohexaose; and (**D**) two-dimensional structure showing the interaction of active site residues and Glu-166, a catalytic residue, with xylohexaose. Purple empty circles are carbon in an xylose ring, O in the purple circle is the sign of oxygen, and the small black circle within the purple circle is the hydrogen. The conventional hydrogen bond (green) and carbon–hydrogen (light green) bond distances show strong interactions between ligands and active site residues. (**E**) shows the distance in angstrom between catalytic residues (Glu-166 and Glu-373).

The sequence alignment of TfGH10 revealed highly conserved amino acid residues, highlighted with a red background, consistent with those in *N. crassa* and other characterized *GH10* genes. All *TfGH10* genes, except *TfGH10-3*, contained the critical catalytic residues Glu (E), essential for xylanase activity. Specifically, Glu catalytic residues were marked with stars in the alignment (Supplementary Figure S2). The binding affinities of TfGH10-4 with ligands X4 and X6 were −8.6 and −9.8, respectively (Table 2), indicating robust interactions with the protein’s 3D pocket-binding sites and catalytic residues (Figure 4A,C). The protein 2D models showed that the catalytic residue Glu-273 interacted zwith the X4 ligand (Figure 4B), while Glu-166 interacted with the X6 ligand (Figure 4D). The distance between Glu-166 and Glu-273 was measured at 5.4 Å, suggesting that these residues acted as strong acids facilitating xylanase activity in *T. fergusii* (Figure 4E).

**Figure 5:**
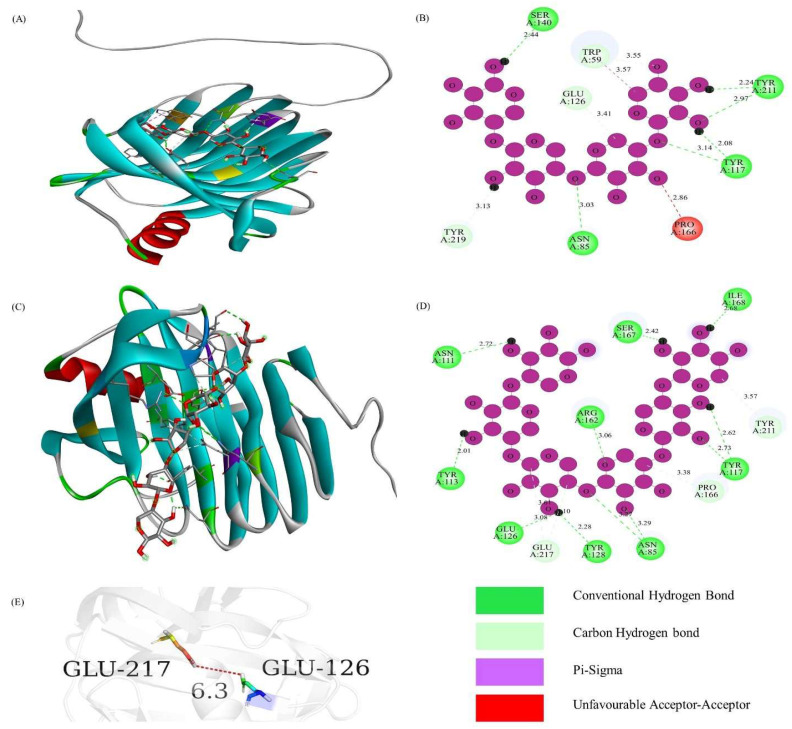
A 3D and 2D structural analysis of TfGH11-1 and its interaction with the ligand: (**A**) 3D structure interaction with xylotetraose; (**B**) 2D structure showing the interaction of active site residues and Glu-126, a catalytic residue, with xylotetraose; (**C**) 3D structure interaction with xylohexaose; (**D**) 2D structure showing the interaction of active site residue Glu-166 and Glu-217, catalytic residue, with xylohexaose; and (**E**) the distance in angstrom between catalytic residues (Glu-126 and Glu-217).

**Figure 6:**
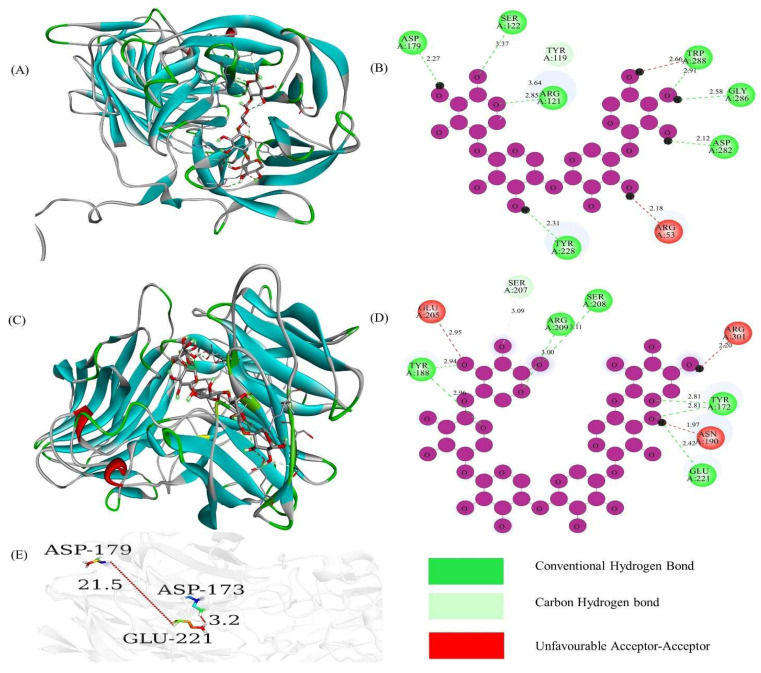
The 3D and 2D structural analysis of TfGH43-6 and its ligand interactions: (**A**) 3D structure interaction with xylotetraose; (**B**) 2D structure showing the interaction of active site residues and Asp-179, a catalytic residue, with xylotetraose; (**C**) 3D structure interaction with xylohexaose; (**D**) 2D structure showing the interaction of active site residues and Glu-221, a catalytic residue, with xylohexaose; and (**E**) the distance in angstrom between catalytic residues (Asp-173, Asp-179, and Glu-217). Here, we show part of the GH43 sequence, and the sequence length of each protein has a significant difference.

Similarly, the sequence alignment of TfGH11 identified conserved residues highlighted with a red background, including the catalytic residues of Glu, which were marked with arrows (Supplementary Figure S3). The binding affinities of TfGH11-1 with ligands X4 and X6 were −9.5 and −9.9, respectively (Table 2), indicating strong interactions with the protein’s 3D pocket-binding sites and catalytic residues (Figure 5A,C). The 2D protein models demonstrated that the catalytic residue Glu-126 formed a carbon–hydrogen bond with the X4 ligand (Figure 5B). At the same time, both Glu-126 and Glu-217 interacted with the X6 ligand via conventional hydrogen bonds and carbon–hydrogen bonds, respectively (Figure 5D). The distance between Glu-126 and Glu-217 was 6.3 Å, underscoring their potential role in xylanase activity (Figure 5E). In contrast, the sequence alignment of TfGH43 showed fewer conserved residues than TfGH10 and TfGH11, likely due to the sequence diversity within the GH43 family. The catalytic residues Asp-222, Asp-228, and Glu-287 were marked with stars (Supplementary Figure S4). The binding affinities of TfGH43-6 for ligands X4 and X6 were −8.3 and −8.7, respectively (Table 2), indicating strong interactions with the protein’s 3D pocket-binding sites and catalytic residues (Figure 6A,C). The 2D protein models revealed that the catalytic residue Asp-179 interacted with the X4 ligand via a conventional hydrogen bond (Figure 6B), while Glu-221 interacted with the X6 ligand through a conventional hydrogen bond (Figure 6D). The distance between Asp-179 and Glu-221 was measured at 21.5 Å, while Asp-173 was closer to Glu-221, with a distance of 3.2 Å (Figure 6E). These findings suggest that Asp-179 may not act as a strong base for TfGH43-6 compared to Asp-173, but it did not interact with the ligand. Further characterization of these genes is necessary to confirm their potential interactions with the substrate.

### 3.5. Gene Duplication, Scaffold Localization, and Ka/Ks Calculation

**Figure 7:**
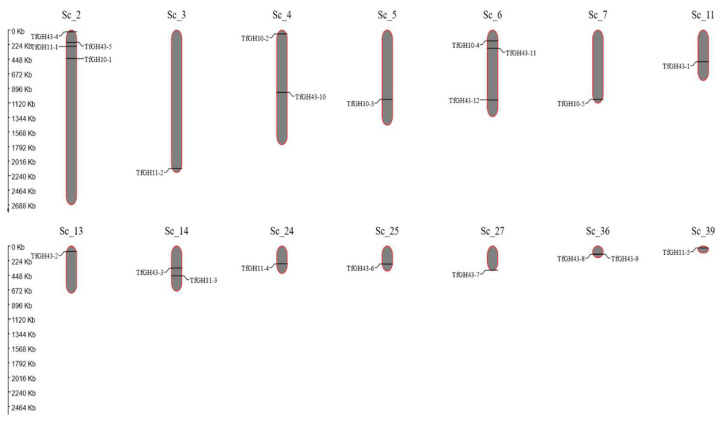
Presentation of TfGH10, TfGH11, and TfGH43 genomic position on the various scaffolds in *T. fergusii*. Scaffold numbers are shown on the upper side of each Sc, and the gene names are listed on both sides of the scaffold.

**Figure 8:**
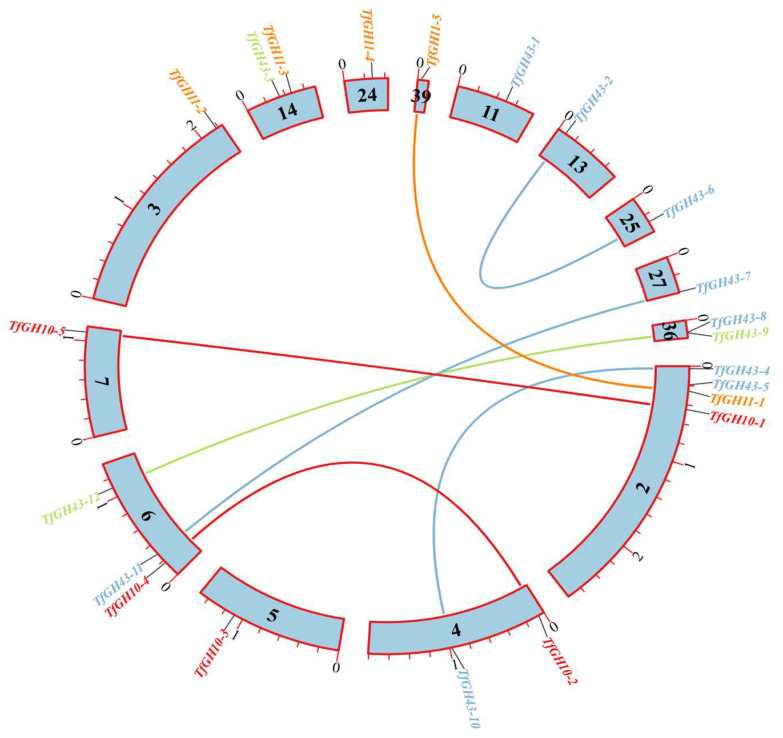
Duplicated genes on the scaffold represented on Circos in *T. fergusii* genome. The different line colors were set based on the gene color of the phylogenetics in the gene structure analysis. One small stick means 0.1 million bp (base pair), while a big stick means 1 million bp on each scaffold.

The xylanase genes were distributed across 14 scaffolds (Sc). Sc_2 and Sc_6 contained the highest number of xylanase genes, with four and three, respectively. Sc_4, Sc_14, and Sc_36 each harbored two genes, while Sc_3, Sc_5, Sc_7, Sc_11, Sc_13, Sc_24, Sc_25, Sc_27, and Sc_39 each contained a single xylanase gene (Figure 7). The Ka/Ks ratio was calculated to evaluate selection pressures, revealing that all gene duplication events were segmental duplications (Supplementary Table S4). Evolutionary analysis indicated that these genes were subjected to different types of selection pressures: Ka/Ks < 1 corresponded to purifying selection, Ka/Ks = 1 represented neutral selection, and Ka/Ks > 1 indicated positive selection (Supplementary Table S4). A collinearity analysis investigated gene duplication within the xylanase gene family. Seven gene duplication pairs were identified, comprising two pairs from the TfGH10 family, one from the TfGH11 family, and four from the TfGH43 family (Figure 8). The Ka/Ks ratio analysis revealed that three duplicated pairs were under purifying selection. The remaining four pairs exhibited Ka/Ks ratios greater than 1, suggesting that these genes were under positive selection (Supplementary Table S4).

### 3.6. Xylanase GO Enrichment and Catalytic Reaction Models

**Figure 9:**
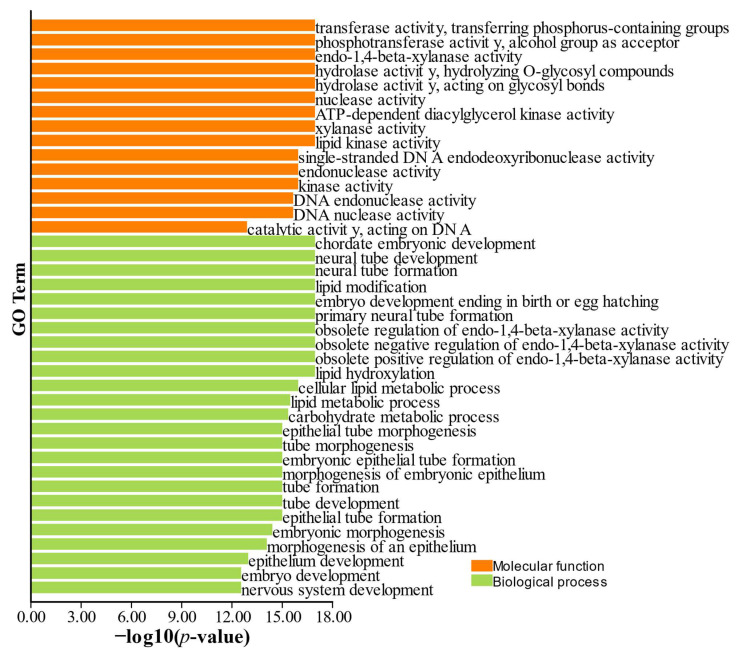
Predicted GO (gene ontology) enrichment of *TfGH10*, *TfGH11*, and *TfGH43* genes. GO associations graph was constructed from the −log *p*-value.

GO enrichment analyses were performed to classify the xylanase genes into two functional categories: molecular function (MF) and biological process (BP) (Supplementary Table S5). The GO-MF analysis revealed the highest enrichment score (181.182) for terms associated with enzymatic activities such as endo-1,4-beta-xylanase and xylan 1,4-beta-xylosidase. Likewise, in the GO-BP category, the 1,2-diacyl-sn-glycero-3-phosphocholine metabolic process exhibited a high enrichment score (164.227) (Supplementary Table S5). The GO-MF enrichment, assessed through -log *p*-values, highlighted key terms, including endo-1,4-beta-xylanase activity (GO:0031176), hydrolase activity targeting O-glycosyl bonds (GO:0004553), and xylanase activity (GO:0097599) (Figure 9).

**Figure 10:**
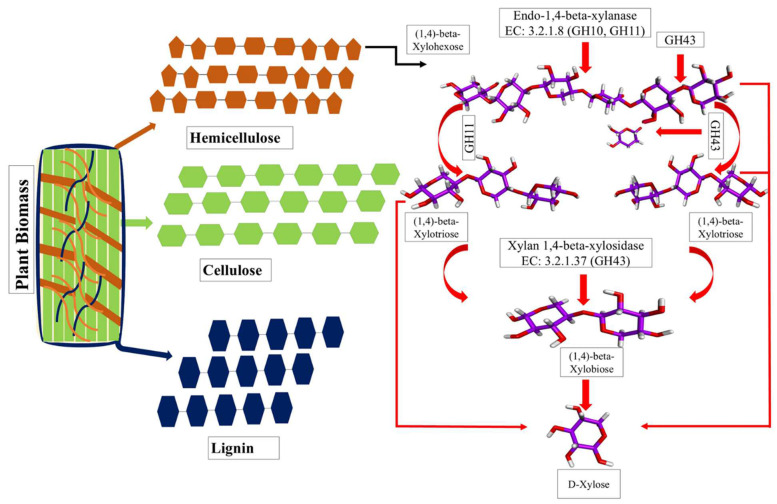
Schematic representation of lignocellulosic biomass composition. Blue represents lignin, green represents cellulose, and dark brown represents hemicellulose. Proposed model of xylanase-mediated degradation of xylohexaose by GH10, GH11, and GH43 enzymes. Lignocellulose is a complex biomass structure, and its deconstruction requires various enzymes for effective hydrolysis. The proposed model shows how GH10 and GH11 xylanase randomly cleavage in a β-1,4 linkage in a complex xylan, with GH11 preferentially acting on linear xylan. GH43 xylanase, which contains complex non-catalytic domains (CBM35, CBM42, and CBM91), plays a role in hydrolyzing debranches and side-chains of hemicellulose. Additionally, GH43 also acts on the non-reducing end of sugar, as shown in this figure.

The catalytic mechanisms of the GH10 and GH11 enzymes involve the cleavage of internal β-1,4-glycosidic bonds within the xylan backbone, resulting in the generation of shorter xylo-oligosaccharides. The GH10 and GH11 enzymes exhibit broad substrate specificity, allowing them to act on a variety of substituted xylan substrates, whereas the GH11 enzymes demonstrate a preference for linear xylan, predominantly producing smaller products such as xylobiose or xylose due to their substrate-specific activity and narrower active site architecture. In contrast, the GH43 enzymes predominantly act on the non-reducing ends of xylan or xylo-oligosaccharides, where the anomeric carbon is engaged in glycosidic bonds, preventing further cleavage at that site. Depending on their structural variations and active site configuration, the GH43 enzymes hydrolyze arabinoxylan side-chains or release monomers such as xylose or arabinose as the final products. These differences in substrate preference and cleavage specificity across the GH10, GH11, and GH43 families highlight their complementary roles in the efficient depolymerization of hemicellulose (Figure 10).

### 3.7. Analysis of Biochemical Properties in Response to Hemicellulose Sugar

**Figure 11:**
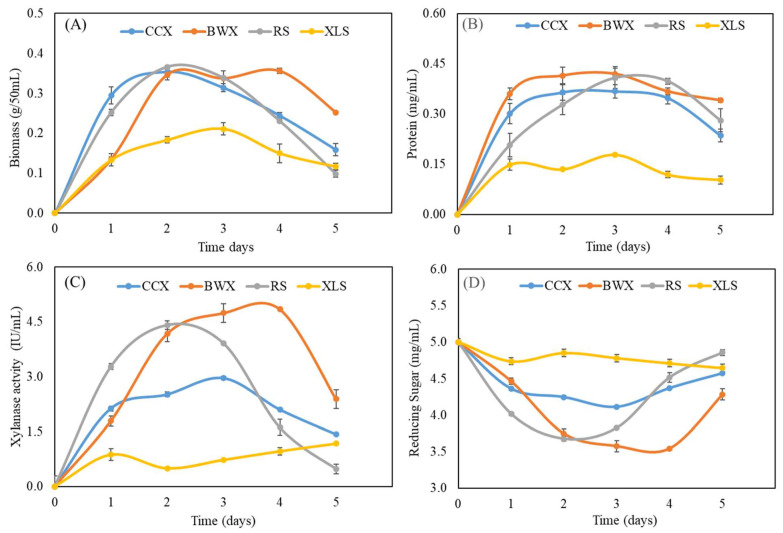
*Thermothelomyces fergusii* cultivated in fermentation medium amended with 2% corncob xylan, beechwood xylan, rice straw, and xylose for five days, and the samples were taken every day to evaluate the biochemical activity at different time points: (**A**) analysis of biomass; (**B**) protein concentration; (**C**) xylanase activity; and (**D**) reducing sugar.

The genome of *T. fergusii* encodes various xylanase enzymes that play a crucial role in xylan degradation and biomass utilization. Temporal biomass measurements revealed a significant increase during the initial three days of cultivation across all substrates (CCX, BWX, RS, and XLS). However, the biomass levels reduced on the fourth and fifth day, except for BWX, which maintained a higher biomass yield on the fourth day than the other substrates. In particular, the lowest biomass production was observed for XLS, a monosaccharide, relative to the polysaccharide substrates (Figure 11A). Similarly, the protein concentrations increased consistently from day 1 to day 3 for all substrates and then decreased on days 4 and 5. BWX supported the highest protein production among the tested substrates, followed by RS, CCX, and XLS (Figure 11B). A comparative analysis of the protein-inducing potential of the substrates revealed significant variations in xylanase secretion, with 2% concentrations of BWX, RS, CCX, and XLS displaying distinct and substrate-specific patterns of enzyme induction. Xylanase activity was quantified using 1% xylan as a substrate at 50 °C for 20 min, with reducing sugar levels measured as an indicator of enzymatic performance. BWX exhibited the highest xylanase induction and activity from days 1 to 4. RS and CCX supported increased xylanase production during the first three days, followed by a reduction on days 4 and 5. In contrast, XLS demonstrated a gradual increase in enzyme secretion and activity over time, although the activity levels were substantially lower than CCX, BWX, and RS (Figure 11C).

Furthermore, the concentration of reducing sugars was positively correlated with enzyme production across all substrates. The complex polysaccharides BWX, RS, and CCX imposed more significant metabolic stress on the xylanase gene network, stimulating higher enzyme production during the initial three days to facilitate efficient biomass degradation. This robust enzymatic response highlights the potential of the xylanase cocktail for industrial applications (Figure 11D).

### 3.8. Gene Expression Analysis Under Hemicellulose Cultivation Conditions

**Figure 12:**
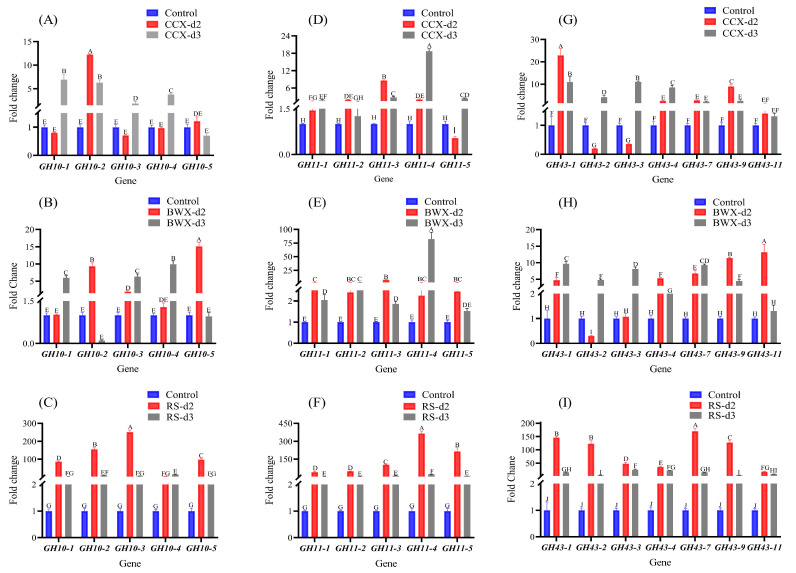
Xylanase gene analysis on the second and third day of cultivation in fermentation medium amended with 2% corncob xylan, beechwood xylan, rice straw, and xylose. Xylose was used as a control to analyze the expression data, and the *GAPDH* gene was used to normalize the data. A statistical analysis was computed with the help of one-way ANOVA analysis in Graphpad-9 software, and standard deviations on each bar were calculated from the three replications in experimental data. Letters on the bars indicate significant differences between the control and treated samples on day 2 and day 3. Gene expression on CCX (**A**) TfGH10, (**D**) TfGH11, and (**G**) TfGH43. Gene expression on BWX (**B**) TfGH10, (**E**) TfGH11, and (**H**) TfGH43. Gene expression on RS (**C**) TfGH10, (**F**) TfGH11, and (**I**) TfGH43.

RT-qPCR is an accurate method for quantifying the expression analysis of target genes. This study analyzed five *TfGH10*, five *TfGH11*, and seven *TfGH43* genes using qPCR. Samples were collected on the second and third days under xylose; a monosaccharide was used as the control gene expression. The results demonstrated that the proportion of upregulated genes was higher on the third day compared to the second day for the *TfGH10*, *TfGH11*, and *TfGH43* genes when cultured on CCX (Figure 12A,D,G). Similarly, on the second day of cultivation with BWX, three *TfGH10*, all five *TfGH11*, and five *TfGH43* genes showed upregulation. On the third day, three *TfGH10*, all *TfGH11*, and all *TfGH43* genes also showed upregulation (Figure 12B,E,H). Interestingly, under RS, the expression levels of all *TfGH10*, *TfGH11*, and *TfGH43* genes were significantly elevated on both the second and third days, with the predominantly highest expression levels observed on the second day for nearly all genes (Figure 12C,F,I).

## 4. Discussion

Research on thermostable xylanases encoded by fungi continues to receive persistent attention, as these enzymes are infrequent but offer considerable industrial potential. The primary objective of this study was to identify industrially relevant xylanases from the genome of the thermophilic fungus *T. fergusii*. Phylogenetic analysis elucidated the evolutionary relationships between the xylanase genes in *T. fergusii* and other filamentous and thermophilic fungi. Many *GH10*, *GH11*, and *GH43* genes in these species have been functionally characterized, demonstrating significant potential for xylan degradation and exhibiting incredible enzymatic activity [13,17,44]. Conserved-domain analysis revealed that the *TfGH10* and *TfGH11* genes contained their respective glycoside hydrolase domains. In contrast, the TfGH43 proteins exhibited significant variability, constituting the second non-catalytic domain, potentially accounting for their unique functional activities with different arabinoxylan substrates.

The GH43 family, often coupled with a CBM35 domain, acts as an α-L-arabinofuranosidase, and it is rarely functionally characterized in fungi, which enhances enzyme efficiency by improving substrate binding and stability, as seen in *Paenibacillus xylanexedens* CBM32, which is functionally similar to CBM35. The dual-domain structure facilitates the hydrolysis of complex polysaccharides, especially in β-1,3 linkage and galactose formation, highlighting these enzymes’ potential role in lignocellulose degradation and biotechnological applications [45]. Moreover, GH43_CBM42 was functionally characterized by *Streptomyces avermitilis*, which potentially hydrolyzes the 1,5 arabinan debranched structure and also acts on *p*-nitrophenol (PNP)-α-l-arabinofuranoside [46]. Similarly, CBM91 present in the C-terminal of GH43 proteins in *Paenibacillus physcomitrellae* plays an important role in hydrolyzing (PNP)-*β*-D-xylopyranoside, xylobiose, and other xylan-related substrates [10].

The analysis identified conserved motifs and consensus residues, including the general base Asp and general acid Glu, which are likely crucial catalytic residues for xylanase activity. Further investigation of the TfGH43 protein sequence revealed two critical motifs containing Asp and Glu residues, which were identified as key catalytic residues in this enzyme family [14]. Structural analysis (3D) of the protein sequences from *T. fergusii* revealed a high degree of similarity with the xylanases from *M. thermophila*, suggesting an evolutionary relationship. In particular, several of these genes were upregulated when *M. thermophila* was cultivated on monocot and dicot plant biomass, further underscoring the significance of *T. fergusii* xylanase activity [47]. Detailed analysis of specific residues in TfGH10-4, TfGH11-1, and TfGH43-6 revealed the presence of conserved catalytic residues, integral to their enzymatic functions (Figure 4, Figure 5 and Figure 6). These findings align with the general acid–base catalytic mechanism proposed for xylanases (EC: 3.2.1.8), wherein Glu acts as a proton donor to the glycosidic oxygen, facilitating bond cleavage, while Asp functions as a nucleophile, coordinating with a water molecule to deprotonate and complete hydrolysis [16,48]. Such catalytic residues have been characterized in multiple xylanase genes, including those in *N. crassa*, *A. niger* An76, and other GH10, GH11, and GH43 proteins, suggesting the functional importance of these genes in *T. fergusii* [13,16,49].

Moreover, specific binding interactions were observed in the TfGH10-4, TfGH11-1, and TfGH43-6 enzymes with X4 and X6 (Figure 4, Figure 5 and Figure 6). We found the interaction of Arg, Asn, Gln, Tyr, and Ser with X4 and X6 in *T. fergusii*, and the role of these residues has been functionally studied in GH11 enzymes from *A. fumigatus* and *A. niger* An76 [49,50]. In addition, the interaction of Tyr residues was more frequent with the X4 and X6 in TfGH11-1 and TfGH43-6 compared to TfGH10-4, which suggests that these residues are binding sites to the substrates. Previous studies also found that Tyr residues bind to the xylose unit of xylan in the binding cleft of the xylanase gene in *Aspergillus kawachii* and many other species [51]. Additionally, GH10 and GH11 enzymes have been shown to release X2 and X3 xylose units by hydrolyzing X6, as detected by thin-layer chromatography (TLC) in *N. crassa* [13]. In contrast, GH11 released X2 and X4 xylose units from X6, as identified by fluorescence-assisted carbohydrate electrophoresis (FACE) in *A. niger* An76 [49]. These insights contribute to a deeper understanding of the evolutionary dynamics and structural features of the GH10, GH11, and GH43 gene families across various fungal species.

Moreover, the different copies of *TfGH10*, *TfGH11*, and *TfGH43* genes and their duplication were observed on different chromosomes of *T. fergusii*. Gene duplication plays a critical role in expanding gene families, allowing for functional diversification and adaptation to ecological niches. The evolution of fungal genomes has been shaped by the expansion of these families, particularly those involved in substrate degradation, which allows fungi to exploit a wide range of environments and organic compounds [52]. In addition, the term molecular function based on GO analysis identified key terms associated with lignocellulose degradation. Similarly, in the transcriptomic analysis of a superior biomass-degrading strain of *A. fumigatus*, GO analysis also identified these key functional categories of genes involved in lignocellulose degradation [53]. In addition, the dominant GO term (0016798) was also analyzed in the transcriptomic analysis of *Trichoderma reesei* [54]. These GO terms reflect the unique function of xylanase genes in different fungal species under different environmental conditions.

Rice straw is an abundant agricultural byproduct with great potential as a feedstock for biorefineries. Its high cellulose and hemicellulose content makes it ideal for reducing sugars, which can be converted into bioethanol and other valuable bio-based products [55]. Xylanase activity assays, combined with protein quantification and secretome profiling, demonstrated that *T. fergusii* efficiently secretes xylanase enzymes when exposed to complex polysaccharide substrates compared to xylose, a mono-sugar. Similarly, the secretome of other thermophilic fungi, such as *Mycothermus thermophilus* and *C. thermophilum*, was enriched with carbohydrate-active enzymes (CAZymes), with xylanase activity being significantly enhanced when xylan was used as the inducing substrate [41,56]. Moreover, *Malbranchea pulchella* GH10 exhibited optimal activity across a broad temperature and pH range when birchwood xylan was used as a substrate [57]. Additionally, *A. niger* An76 exhibited increased xylanase activity over time when cultured on xylan [58]. Significantly, when *M. thermophila* was grown on pretreated rice straw, its xylanase activity and reducing sugar increased over time [59]. During the initial 1-3 days of incubation under shaking conditions, *T. fergusii* demonstrated a relatively dry biomass yield compared to *C. thermophilum* and *A. niger* An76. Moreover, enzymatic activity and protein concentrations peaked on days 2 and 3 of induction across various xylan substrates (Figure 11). Similarly, the synergistic action of GH11 xylanase (RrXyn11A) and GH43 xylosidase (RrXyl43A) from *Rhizophlyctis rosea* significantly enhanced the degradation of wheat bran and beechwood xylan, highlighting their potential for efficient biomass hydrolysis [60].

The secretome analysis of *T. fergusii* revealed a significant enrichment of xylanase enzymes, as demonstrated by their robust enzymatic activity. Transcript expression profiling further indicated that the expression of xylanase-encoding genes (*TfGH10*, *TfGH11*, and *TfGH43*) was strongly upregulated when the fungus was cultivated on a complex substrate (Figure 12). Comparative expression analysis with other thermophilic fungi further supports these findings. In *C. thermophilum*, a GH10 xylanase containing a CBM1 domain was co-expressed with key cellulases, indicating its role in synergistic lignocellulose degradation [41]. Moreover, in *N. crassa*, cultivation in a xylan-based medium induced the upregulation of all predicted xylanolytic enzymes [13]. Similarly, *M. cinnamomea* exhibited substantial upregulation of GH10, GH11, and GH43 xylanases when exposed to xylan, compared to glucose, further emphasizing the role of these genes in hemicellulose breakdown [22]. In addition, RT-qPCR analysis in *Paenibacillus physcomitrellae XB* revealed that two GH43 genes were upregulated on CCX and xylose, encoding bifunctional β-xylosidase/α-L-arabinofuranosidase enzymes. Screening and expression analysis confirmed their strong activity in hydrolyzing CCX (xylo-oligosaccharides), with Ppxyl43B exhibiting greater stability, likely due to its extra GH43_C2 domain, making these enzymes promising for xylan biomass conversion [9]. In *T. terrestris* LPH172, transcriptomic data revealed a significant induction of multiple xylanase genes in response to RS and BWX, highlighting conserved regulatory mechanisms across thermophilic fungi and other microorganisms [61].

The efficient degradation of lignocellulosic biomass for biofuel and biorefinery applications remains challenging due to its complex structure and the high cost of enzymatic hydrolysis [62]. Unlike cellulose, hemicellulose is more heterogeneously distributed, making xylan–enzyme interactions crucial for improving biomass deconstruction [63]. A diverse repertoire of xylanase genes in *T. fergusii* highlights the need for multi-enzyme cooperation in biomass conversion. Carbohydrate-binding modules (CBMs) significantly influence xylanase efficiency and substrate specificity. In *Aspergillus fumigatus* Z5, a GH10 xylanase containing CBM1 showed reduced activity on pure xylan but enhanced hydrolysis on washed corncob particles (WCCPs), while CBM1-lacking GH11 xylanases remained active on WCCPs [11]. Similarly, transcriptomic analyses in *C. thermophilum* showed CBM1-containing GH10 xylanases co-expressed with cellulases, suggesting a synergistic function for biomass degradation [41]. These findings underscore the structural importance of xylanases in substrate adaptation. Comparative structural analysis of *T. fergusii* revealed 14 xylanases with strong homology to known xylanases in *M. thermophila*. Functional studies in *M. thermophila* demonstrated that Xyr1 deletion abolished growth on xylan and xylose [64]. Sequence alignment of TfGH10 and TfGH11 with *N. crassa* revealed conserved residues across GH10, GH11, and GH43 families. Deletion of gh10-1, gh10-2, and gh43-1 in *N. crassa* reduced growth on xylan, underscoring the conserved role of these genes in hemicellulose degradation [13]. Substrate complexity further influenced xylanase expression. In *M. cinnamomea*, wheat bran, a complex substrate, induced broader CAZyme expression than beechwood xylan [22]. Similarly, in *T. fergusii*, the *TfGH10*, *TfGH11*, and *TfGH43* genes were upregulated on the second and third days of rice straw induction, demonstrating their adaptability to diverse biomass sources and their potential for industrial lignocellulose conversion.

## 5. Conclusions

The genomic and expression analysis of *T. fergusii* revealed a diverse repertoire of xylanase genes, including *GH10*, *GH11*, and *GH43*, that exhibited significant upregulation in response to complex lignocellulosic substrates, such as rice straw, beechwood xylan, and corncob xylan. Substantially, multiple carbohydrate-binding modules (CBMs) in these enzymes suggest a highly adaptive lignocellulose degradation system. CBM1 facilitates cellulose binding, enhancing the accessibility of enzymatic interaction, while CBM35, CBM42, and CBM91 contribute to the degradation of arabinoxylans by targeting side-chain modifications and debranching arabinose substitutions. This synergistic enzyme cocktail in *T. fergusii* underscores its potential as a highly efficient system for lignocellulosic biomass degradation, offering valuable insights into biotechnological applications in biofuel and biorefinery industries. In our future study, functional and structural characterization of these xylanases will deepen our understanding of their catalytic efficiency and substrate specificity, paving the way for their optimized application in biomass valorization, biofuel production, and other biotechnological processes.

## Figures and Tables

**Table 1:** Detailed information on TfGH10, TfGH11, and TfGH43 proteins’ properties in *Thermothelomyces fergusii*.

Original Gene ID	Gene ID Rename	Scaffold	Second Domain	EC Number	Genomic Position	SP	Gene Length	CDS (bp)	AA (bp)	E:I	SI%	SS%
gene_3198	TfGH10-1	scaffold_2	-	3.2.1.8	446,763–448,277**−**	17	1515	1068	356	4:3	76.9	87.6
gene_6450	TfGH10-2	scaffold_4	-	3.2.1.8	59,346–60,446**+**	19	1101	1101	367	1:0	78.6	85.4
gene_7390	TfGH10-3	scaffold_5	-	3.2.1.8	1,092,416–1,093,212**+**	15	797	510	170	4:3	44.6	47.7
gene_7581	TfGH10-4	scaffold_6	-	3.2.1.8	169,430–170,763**+**	18	1334	1122	374	3:2	87.2	93.6
gene_8290	TfGH10-5	scaffold_7	CBM1	3.2.1.8	1,092,564–1,093,928**+**	17	1365	1146	382	4:3	84.8	90.4
gene_3142	TfGH11-1	scaffold_2	-	3.2.1.8	254,904–256,034**+**	16	1131	693	231	2:1	94.8	96.5
gene_5897	TfGH11-2	scaffold_3	-	3.2.1.8	2,178,512–2,179,256**+**	18	745	666	222	2:1	81.9	91.9
gene_2026	TfGH11-3	scaffold_14	-	3.2.1.8	467,894–468,688**−**	20	795	687	229	2:1	88.2	93.4
gene_4601	TfGH11-4	scaffold_24	CBM1	3.2.1.8	277,861–278,860**−**	19	1000	870	290	2:1	73	80.2
gene_6409	TfGH11-5	scaffold_39	-	3.2.1.8	31,507–32,272**−**	16	766	666	222	2:1	94.6	96.9
gene_1275	TfGH43-1	scaffold_11	-	-	496,981–497,996**+**	No	1016	948	316	2:1	55.2	60
gene_1647	TfGH43-2	scaffold_13	CBM91	3.2.1.37	84,744–86,432**−**	24	1689	1689	563	1:0	63.9	74.9
gene_1989	TfGH43-3	scaffold_14	CBM35	-	346,458–347,582**+**	No	1125	1125	375	1:0	74.5	75.1
gene_3073	TfGH43-4	scaffold_2	CBM91	3.2.1.37	25,833–27,446**−**	No	1614	1614	538	1:0	97	99.1
gene_3126	TfGH43-5	scaffold_2	CBM35	-	193,250–195,023**−**	18	1774	1356	452	5:4	90.7	97.8
gene_4738	TfGH43-6	scaffold_25	CBM91	3.2.1.37	282,021–283,700**−**	23	1680	1680	560	1:0	85.9	91.1
gene_5010	TfGH43-7	scaffold_27	-	-	379,672–380,655**+**	No	984	984	328	1:0	86.5	93
gene_6310	TfGH43-8	scaffold_36	-	-	127,171–128,387**−**	19	1217	963	321	4:3	93.1	96.9
gene_6311	TfGH43-9	scaffold_36	-	3.2.1.99	129,332–130,498**+**	21	1167	963	321	3:2	90.3	95
gene_6730	TfGH43-10	scaffold_4	CBM91	-	980,624–982,408**−**	23	1785	1785	595	1:0	81.9	85.8
gene_7609	TfGH43-11	scaffold_6	CBM42	-	286,430–287,869**+**	27	1440	1440	480	1:0	79.7	86.8
gene_7865	TfGH43-12	scaffold_6	-	-	1,100,905–1,102,152**+**	25	1248	1065	355	4:3	91.5	95.5

EC, enzyme commission; SP, signal peptide; CD, coding sequence; AA, amino acid; Genomic positions of xylanase genes, with strand orientation: (**+**) for *5′* to *3′* transcription and (**−**) for *3′* to *5′* transcription relative to the reference genome; E:I, exon–intron ratio; SI, sequence identity, determined based on the pairwise analysis of *T. fergusii* proteins and AlphaFold-predicted protein models; and SS, sequence similarity assessed using the same pairwise approach (Supplementary Table S3).

**Table 2:** Protein–ligand binding affinity.

Gene Name	Xylotetraose (Kcal/mol)	Xylohexaose (Kcal/mol)
TfGH10-4	−8.6	−9.8
TfGH11-1	−9.5	−9.9
TfGH43-6	−8.3	−8.7

## Data Availability

The original contributions presented in this study are included in the article and Supplementary Materials. Further inquiries can be directed to the corresponding author.

## Supplementary Materials

The following supporting information can be downloaded at https://www.mdpi.com/article/10.3390/jof11040250/s1, Figure S1: Putative motif analysis from TfGH10, TfGH11, and TfGH43 proteins using MEME online website; Figure S2: GH10 protein sequence alignment in *M. thermophia* (Mt), *N. crassa* (Nc), *M. cinnamomea* (Mc), and *T. fergusii* (Tf); Figure S3: GH11 protein sequence alignments in *M. thermophia* (Mt), *N. crassa* (Nc), *M. cinnamomea* (Mc), and *T. fergusii* (Tf); Figure S4: GH43 protein sequence alignment in *M. thermophia* (Mt), *N. crassa* (Nc), *M. cinnamomea* (Mc), and *T. fergusii* (Tf); Table S1: GH10, GH11, and GH43 protein sequences in *M. thermophia* (Mt), *N. crassa* (Nc), *M. cinnamomea* (Mc), and *T. fergusii* (Tf); Table S2: Primers used for qPCR expression analysis; Table S3: AlphaFold-predicted protein models; Table S4: Ka/Ks analysis of xylanase gene; and Table S5: gene ontology enrichment analysis.
