# Supplementary material for: Harnessing Xylanase Potential in Thermothelomyces fergusii: Insights from Computational and Functional Analysis
Source: J Fungi (Basel). 2025 Mar 25;11(4):250. doi: 10.3390/jof11040250 (PMC12028744; doi:10.3390/jof11040250)
Supplement: Supplementary file 1 [file jof-11-00250-s001.zip › jof-3521256-supplementary/2.Supplementary Table S2.pdf]

Supplementary Table S2. Primers used for qPCR expression analysis.

Sr. No	Gene Name	Forward Primer	Reverse Primer
1	*TfGH10-1*	CTACCGGGACAACGTCTTC	GTCGTTGTAGTACAGCTTCG
2	*TfGH10-2*	CAGTACGACGCCATCTTC	CCTCGGTGAAGTTGAACAG
3	*TfGH10-3*	CGGAACAACAAGACCATCC	GTAGTCGTTGGTGCTGTG
4	*TfGH10-4*	GCGAGGAGTACATCAAGC	CGAGGTTGTAGTCGTTGTAG
5	*TfGH10-5*	CAAGGGCAAGGTCTACTTC	CCACTTCAGGCTGTTCTC
6	*TfGH11-1*	CGGCAGCAACTACAAGATC	CAGAACTGGTCGAAGGTG
7	*TfGH11-2*	CAATATCCGCTGCAACAAC	CGATTGCCTCCGTAACTC
8	*TfGH11-3*	GCACAACGGCTACTACTAC	CAGTGTTCCTCCAGTTGAC
9	*TfGH11-4*	GTTCTGCTCGCAACATCAC	CGATGTAGTACTCGATCAGC
10	*TfGH11-5*	CGCTGATCGAGTACTACATTG	CGTATAAGTGCCGCCATC
11	*TfGH43-1*	GGTACGTGGCGTACAAGG	CGACCTCCTGCAGCATC
12	*TfGH43-2*	CACCAACTTCTGGATCACC	GCCCGTTGTCGTAGTAAC
13	*TfGH43-3*	GCGTAATCGTCACTGGTATC	CATGTCGTCCGTGTTCTG
14	*TfGH43-4*	CGACTGGCTCTACGTCAAG	CTCGAACGTGTACCTCTTCTC
15	*TfGH43-7*	CAGTACGACATGGTGGACTAC	CCAGAGCTGCTTCGACAC
16	*TfGH43-9*	GCAACTACTTCCTCACCTTC	GTCATAGGCGACCTGGTAC
17	*TfGH43-11*	CATCGGGTACAAGGTCAAC	CATGCCCATGCAGTAGTTG
18	*GAPDH*	GCGTCAACGAGAAGACCTAC	GTGGTCATCAGACCCTCAATG
