# Supplementary material for: Harnessing Xylanase Potential in Thermothelomyces fergusii: Insights from Computational and Functional Analysis
Source: J Fungi (Basel). 2025 Mar 25;11(4):250. doi: 10.3390/jof11040250 (PMC12028744; doi:10.3390/jof11040250)
Supplement: Supplementary file 1 [file jof-11-00250-s001.zip › jof-3521256-supplementary/4. Supplementary Table S4.pdf]

Table S4. Ka/Ks analysis of xylanase gene.

Seq_1	Seq_2	Ka*	Ks*	Ka/Ks	Duplication types
TfGH10-1	TfGH10-5	0.489042	0.485429	1.007442	Segmental
TfGH10-2	TfGH10-4	0.30189	0.424505	0.711158	Segmental
TfGH11-1	TfGH11-5	0.22199	0.473289	0.469037	Segmental
TfGH43-2	TfGH43-6	0.560483	0.53393	1.049732	Segmental
TfGH43-4	TfGH43-10	0.777723	0.759985	1.023339	Segmental
TfGH43-7	TfGH43-11	0.689764	0.564456	1.221997	Segmental
TfGH43-9	TfGH43-12	0.892188	1.637511	0.544844	Segmental

Ka*nonsynonymous; Ks* synonymous
