# Supplementary material for: Harnessing Xylanase Potential in Thermothelomyces fergusii: Insights from Computational and Functional Analysis
Source: J Fungi (Basel). 2025 Mar 25;11(4):250. doi: 10.3390/jof11040250 (PMC12028744; doi:10.3390/jof11040250)
Supplement: Supplementary file 1 [file jof-11-00250-s001.zip › jof-3521256-supplementary/Supplementary Figure S1-S4.pdf]

**1**	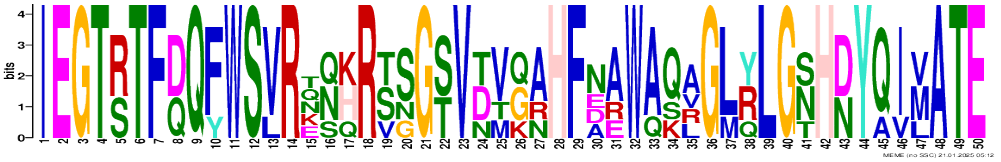 IEGTRTFDQFWSVRENKRTSGSVDVGAHFNAWAQAGLRLGSHDY QIVATE
**2**	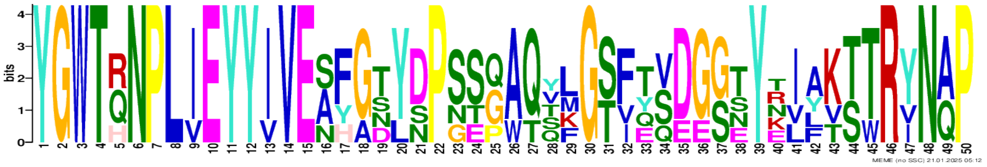 YGWTQNPLIEYYIVEAFGTYDPSSGAQTLGSFTVDGGTYKIYKTT RYNAP
**3**	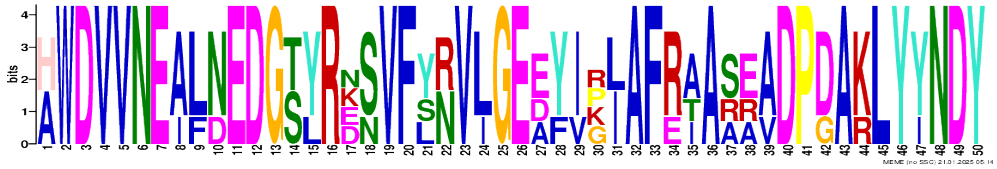 AWDVVNEALNEDGSYRDSVFYRVLGEEYIKJAFRAASEADPDAK LYYNDY
**4**	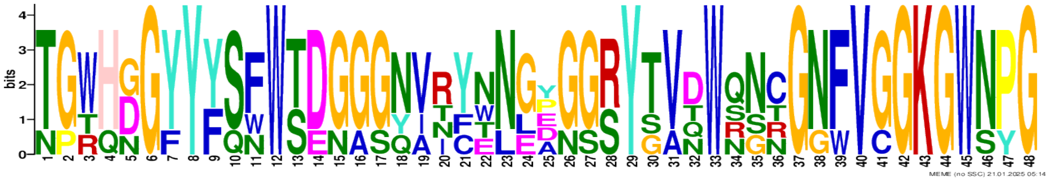 TGWHGGYYYSFWTDGGGNVRYNNGEGGRYTVDWQNCGNFVG GKGWNPG
**5**	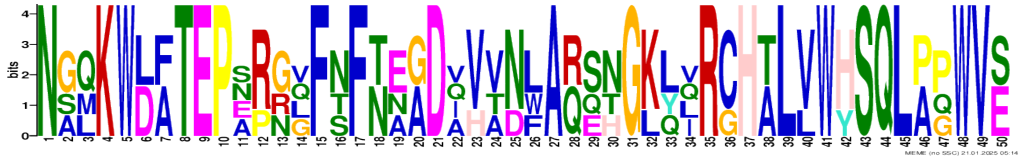 NGQKWDFTEPARGLFNFTEGDVVVNLARSNGKLIRCHALVWHS QLPPWVE
**6**	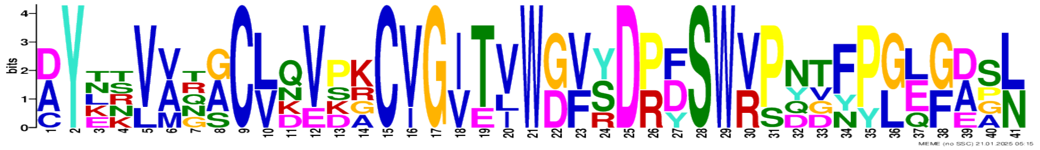 AYKSVVQACLQVPKCVGITVWGVYDPFSWVPNTFPGEGDSL

**7**	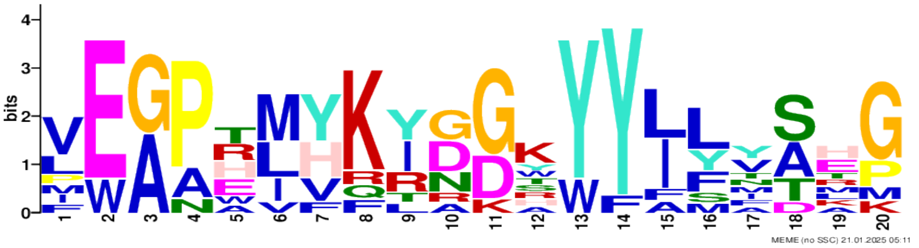<br>VEAPEMYKIDGKYYJLVSEG
**8**	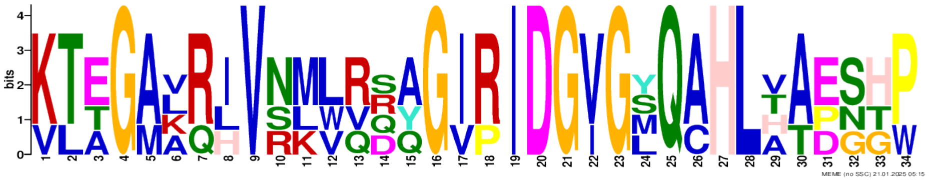<br>KTEGAVRIVNMLRQAGIRIDGVGLQAHLVAESHP
**9**	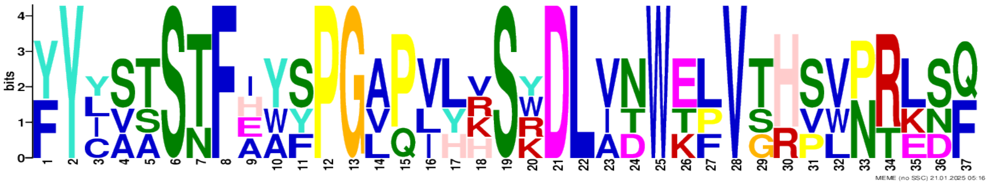<br>YYLSTSTFAYYPGAPVLKSRDLVNWELVTHSVPRLSF
**10**	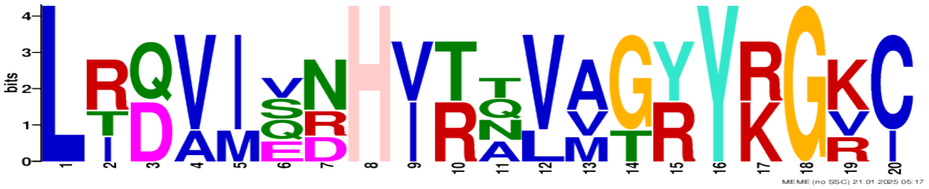<br>LRDVIENHIRTVAGRYKGKC

Supplementary Figure S1. Putative motifs analysis from TfGH10, TfGH11, and TfGH43 proteins using MEME online website.

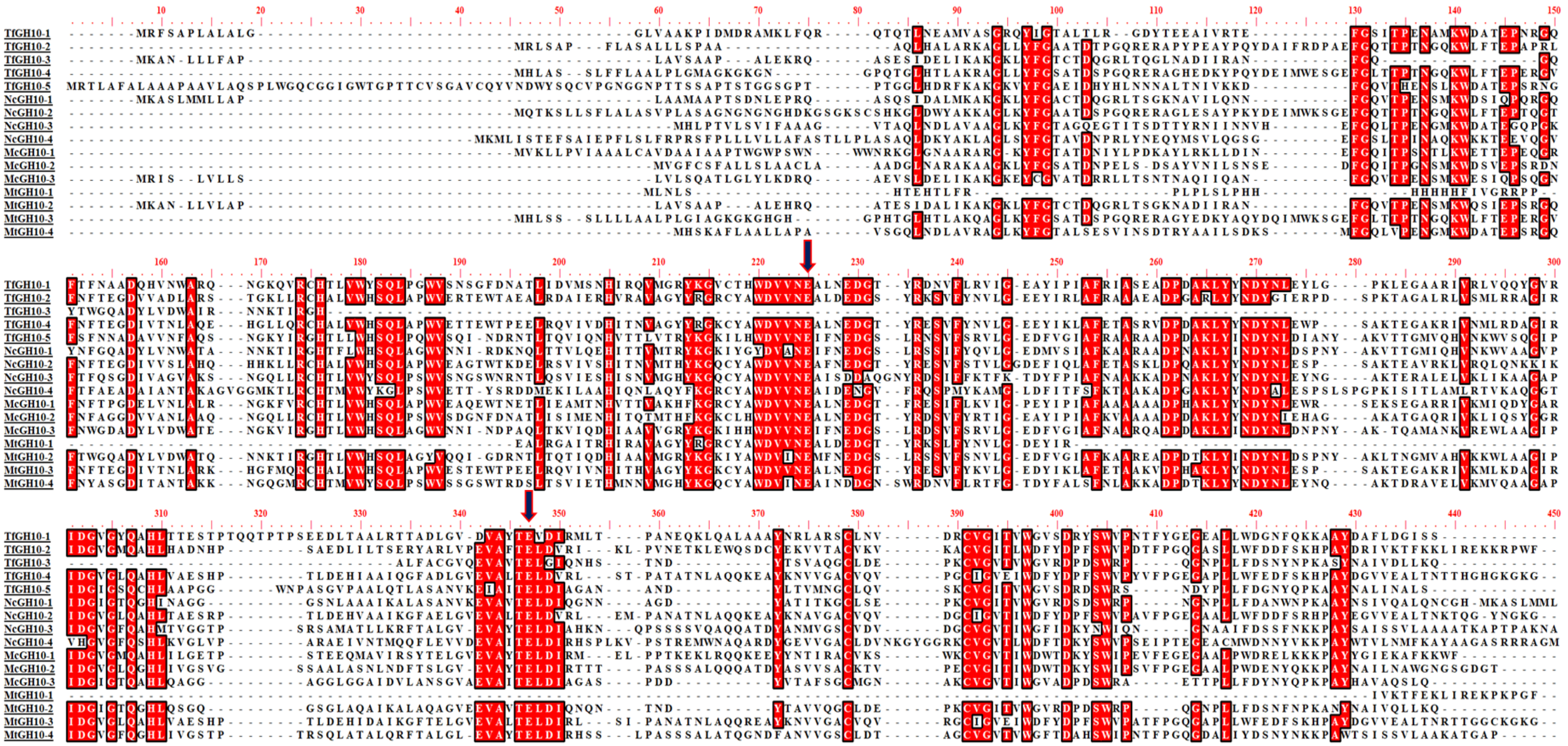

Supplementary Figure S2. GH10 proteins sequence alignment in *M. thermophia* (Mt), *N. crassa (*Nc*), M cinnamomea* (Mc)*,* and *T. fergusii* (Tf): A red background showing conserved residues, two arrows are displaying catalytic residues glutamic acids (Glu/E) among the four species of fungi. Here, we show 450 amino acids for all sequences; some GH10 protein sizes are more than 450 AA.

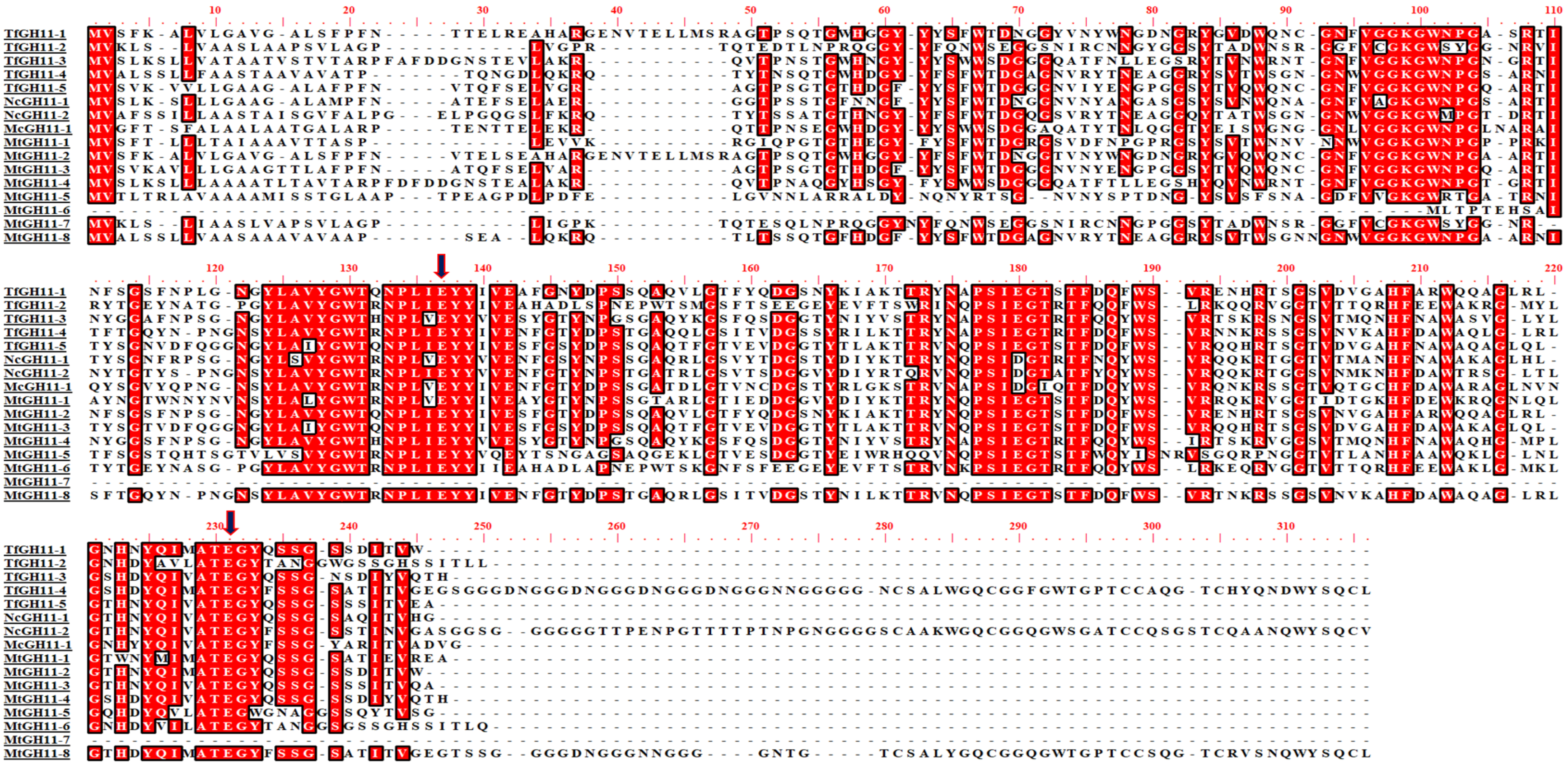

Supplementary Figure S3. GH11 proteins sequence alignments in *M. thermophia* (Mt), *N. crassa (*Nc*), M cinnamomea* (Mc), and *T. fergusii* (Tf): A red background showing conserved residues, two arrows showing catalytic residues glutamic acids (Glu/E) among the four species of fungi.

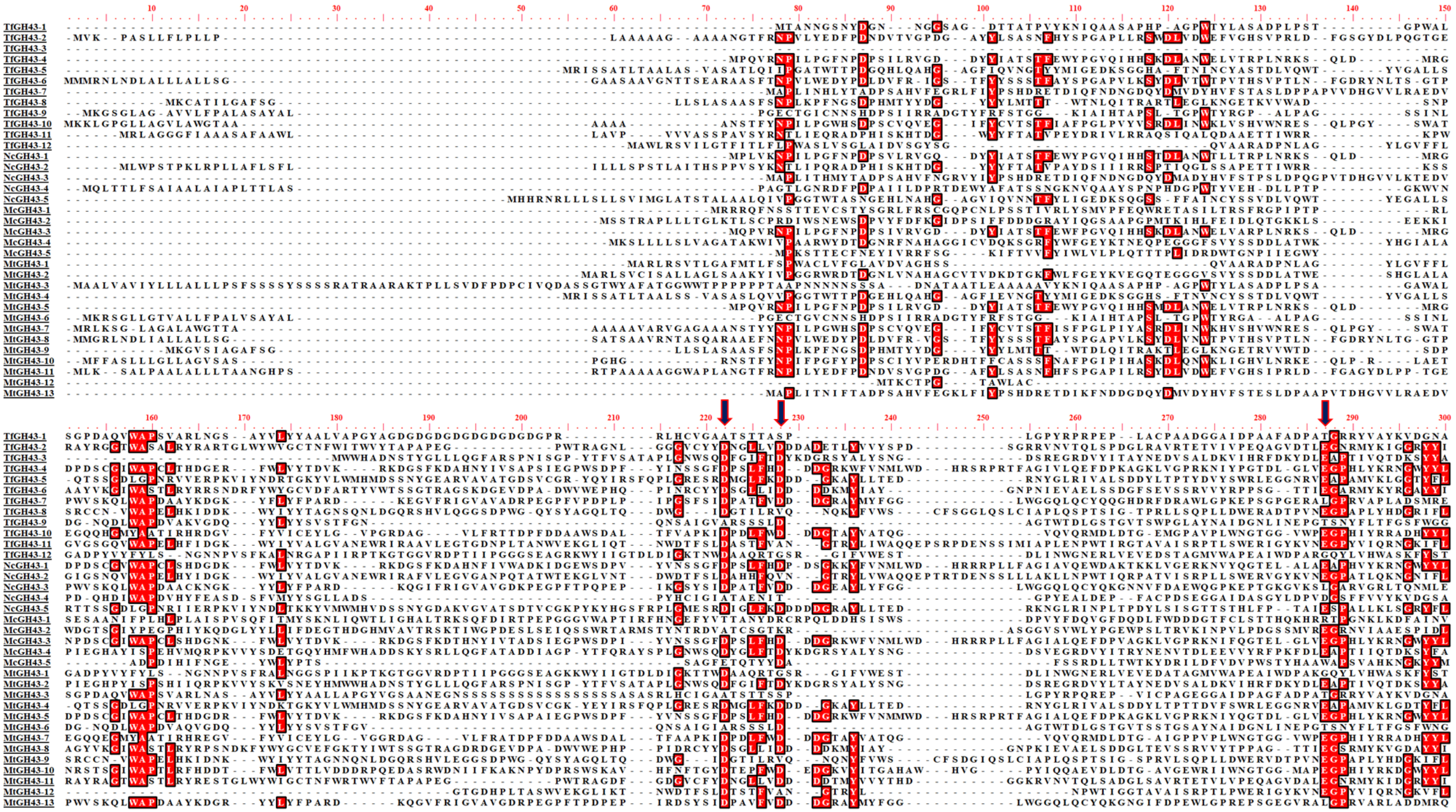

Supplementary Figure S4. GH43 proteins sequence alignment in *M. thermophia* (Mt)*, N. crassa (*Nc*), M cinnamomea* (Mc)*,* and *T. fergusii* (Tf): A red background showing conserved residues, three arrows are displaying catalytic residues including two Aspartic acids (Asp/D) and one glutamic acid (Glu/E) among the four species of fungi. Here, we show 450 amino acids for all sequences; some GH10 protein sizes are more than 600 AA. According to our interaction analysis, Asp-179 residue (D-228 in this figure) interacted with the ligand but interpreting the distance between Glu and Asp-173, then asp-173 (D-222 in this figure) was near to Glu, therefore it is possible that both Asp can perform as a catalytic residue. Functional characterization will clarify the function of these predicted residues.
